# HMA-GCA: hybrid manifold augmentation and gated cross-attention for circRNA-miRNA interaction prediction

**DOI:** 10.1093/bioinformatics/btag536

**Published:** 2026-07-20

**Authors:** Yunzhou Hu, Yansu Wang, Yifeng Bai, Lei Xu, Quan Zou, Hao Zhou, Chunyu Wang, Mengting Niu

**Affiliations:** Institute of Fundamental and Frontier Sciences, University of Electronic Science and Technology of China, Chengdu, China; Yangtze Delta Region Institute (Quzhou), University of Electronic Science and Technology of China, Quzhou, China; Institute of Fundamental and Frontier Sciences, University of Electronic Science and Technology of China, Chengdu, China; Yangtze Delta Region Institute (Quzhou), University of Electronic Science and Technology of China, Quzhou, China; Department of Oncology, Sichuan Provincial People’s Hospital, University of Electronic Science and Technology of China, Chengdu, China; School of Electronic and Communication Engineering, Shenzhen Polytechnic University, Shenzhen, 518055, China; Institute of Fundamental and Frontier Sciences, University of Electronic Science and Technology of China, Chengdu, China; Yangtze Delta Region Institute (Quzhou), University of Electronic Science and Technology of China, Quzhou, China; Department of Stomatology, Sichuan Provincial People’s Hospital, University of Electronic Science and Technology of China, Chengdu, China; Faculty of Computing, Harbin Institute of Technology, Harbin, China; Institute of Fundamental and Frontier Sciences, University of Electronic Science and Technology of China, Chengdu, China; Yangtze Delta Region Institute (Quzhou), University of Electronic Science and Technology of China, Quzhou, China

## Abstract

**Motivation:**

Circular RNAs (circRNAs) interact with microRNAs (miRNAs) to regulate gene expression and influence disease progression. However, traditional models tend to overlook the significant contributions of certain features when dealing with diverse sequence information, resulting in the inability to capture some deep topological structures and thus leaving room for improvement in prediction performance.

**Results:**

We propose HMA-GCA, a novel framework that integrates hybrid manifold augmentation and gated cross-attention for CMI prediction. The model first constructs multi-scale descriptors by combining sequence-derived features (K-mer, CTD, Doc2Vec) and topological features (Role2Vec, node degree, neighborhood proximity). It then applies PCA for global linear projection and UMAP for local nonlinear manifold learning, enhancing feature representations while preserving intrinsic data geometry. A channel-wise gated cross-attention mechanism dynamically controls the injection of miRNA information into circRNA representations. Extensive experiments on three benchmark datasets show that HMA-GCA consistently outperforms state-of-the-art methods across multiple metrics. To ensure interpretability, we conducted SHAP analysis to quantify the contribution of each feature type, revealing that sequence-derived features and topological similarities are the most influential. Ablation studies confirm the necessity of each module, while case studies demonstrate that top-ranked predictions are supported by literature evidence. Overall, HMA-GCA not only achieves state-of-the-art predictive performance but also provides interpretable insights into the molecular features.

**Availability and implementation:**

The source code and data are freely available at https://github.com/Lixunwind/Prediction-circ-mi-by-Gate.git. The implementation is based on Python and the required dependencies are listed in the repository.

## 1 Introduction

Circular RNAs (circRNAs) are a class of endogenous non-coding RNAs characterized by a covalently closed loop structure formed through back-splicing, which confers exceptional stability and enables them to function as efficient microRNA sponges (Memczak *et al*. 2013, Nielsen *et al*. 2022, Liu *et al*. 2024, Xu *et al*. 2024). MicroRNAs (miRNAs) are small non-coding RNAs that post-transcriptionally regulate gene expression by base-pairing with complementary sequences in messenger RNAs (Bartel 2004). CircRNA and miRNA, as important factors among non-coding RNAs, their interaction is closely related to a variety of human diseases (Hansen *et al*. 2013, Kristensen *et al*. 2019, Tong *et al*. 2022, Qiao *et al*. 2024, Luo *et al*. 2025, Niu *et al*. 2025, Zhuang *et al*. 2026). For example, Li *et al.* reported that circPVT1 acts as a competing endogenous RNA that regulates E2F2 expression and tumorigenesis in a miR-125b-dependent manner (Li *et al*. 2018). Similarly, Geng *et al.* demonstrated that circSMARCA5 promotes tumor progression by modulating the miR-17-3p–EGFR axis and may represent a potential therapeutic target for lung adenocarcinoma (Geng *et al*. 2023). These examples underscore the therapeutic and diagnostic potential inherent in understanding circRNA-miRNA interactions (CMIs) across diverse disease contexts. Despite their biological significance, experimental validation of CMIs through traditional wet laboratory approaches remains prohibitively time-consuming, labor-intensive, and costly. This experimental bottleneck has catalyzed the development of computational prediction models capable of efficiently prioritizing high-confidence CMI candidates for subsequent experimental validation.

With the rapid development of big data analysis technologies, the development of computational methods has also been very rapid, evolving from simply calculating correlations through sequence features in the early days to now calculating correlations through multiple approaches (Zhang and Fan 2017, Jiang and Zhu 2020, Jin *et al*. 2022, Ai *et al*. 2024, Liu *et al*. 2024, Wang *et al*. 2024, Momanyi *et al*. 2026). In related miRNA interaction prediction tasks, graph-based models and feature-fusion strategies have also been increasingly explored. For example, GCNCRF combines graph convolutional networks with attention-enhanced conditional random fields to capture heterogeneous network information in lncRNA–miRNA interaction prediction (Wang *et al*. 2022). HetGAT-LMI further introduces heterogeneous graph attention and integrates sequence, structural, and network features to improve multi-relation modeling and interpretability (Liu *et al*. 2026). In addition, DVCH-LMI discusses the influence of negative sampling and class imbalance in lncRNA–miRNA interaction prediction, indicating that sample construction remains an important factor in model training (Liu *et al*. 2025). Beyond RNA interaction prediction, AMPred-LWN also shows that integrating multiple types of features can improve prediction performance in biological prediction tasks (Han *et al*. 2026). These studies indicate that graph modeling, feature fusion, and sample construction have become important concerns in computational biological association prediction. Among these directions, one representative strategy is multimodal stacking. The idea of this method is to extract topological structure features by using graph attention networks, etc., and then operate on the topological structure features to capture the nonlinear correlation properties between circRNA and miRNA. The MUSCLE model proposed by Ji *et al.* adopts this method (Ji *et al*. 2024). The MDA module in it collects topological structure features, and then the MS-CAM module is used to achieve weighted fusion in the high-order feature space. However, although this multi-view design enriches the representation of features, the interaction between different views is still relatively shallow and mainly relies on static low-order representations, which limits the exploration of deep collaborative features between circRNA and miRNA.

Another idea is to use the Multi-view GCN method in feature extraction. This method holds that features cannot be simply concatenated but need to be processed to reduce the mutual interference between different modal features while retaining topological properties (Xu *et al*. 2025). The AMHMDA model constructed by Qiao *et al.* is based on this idea (Ning *et al*. 2023). They introduced Hypergraph and Supernode to handle topological relationships. Nevertheless, although AMHMDA integrates heterogeneous biological information, the aggregation of node features is still largely based on linear combinations of high-dimensional sparse representations, which may overlook the intrinsic manifold structure underlying the feature space and thus fail to fully capture the geometric relationships between samples.

It should be noted that all the models mentioned above employ the Cross-Attention mechanism (Vaswani *et al*. 2017, Chen *et al*. 2025), which means that in actual prediction, the features of circRNA or miRNA cannot be viewed in isolation. Only by implementing dynamic interactive modeling can the final result be improved. In addition, conventional cross-attention mechanisms usually rely on global dot-product similarity, which may miss fine-grained channel-level correlations and lack explicit gating mechanisms to control information flow between modalities, potentially introducing noise from irrelevant features.

**Figure 1:**
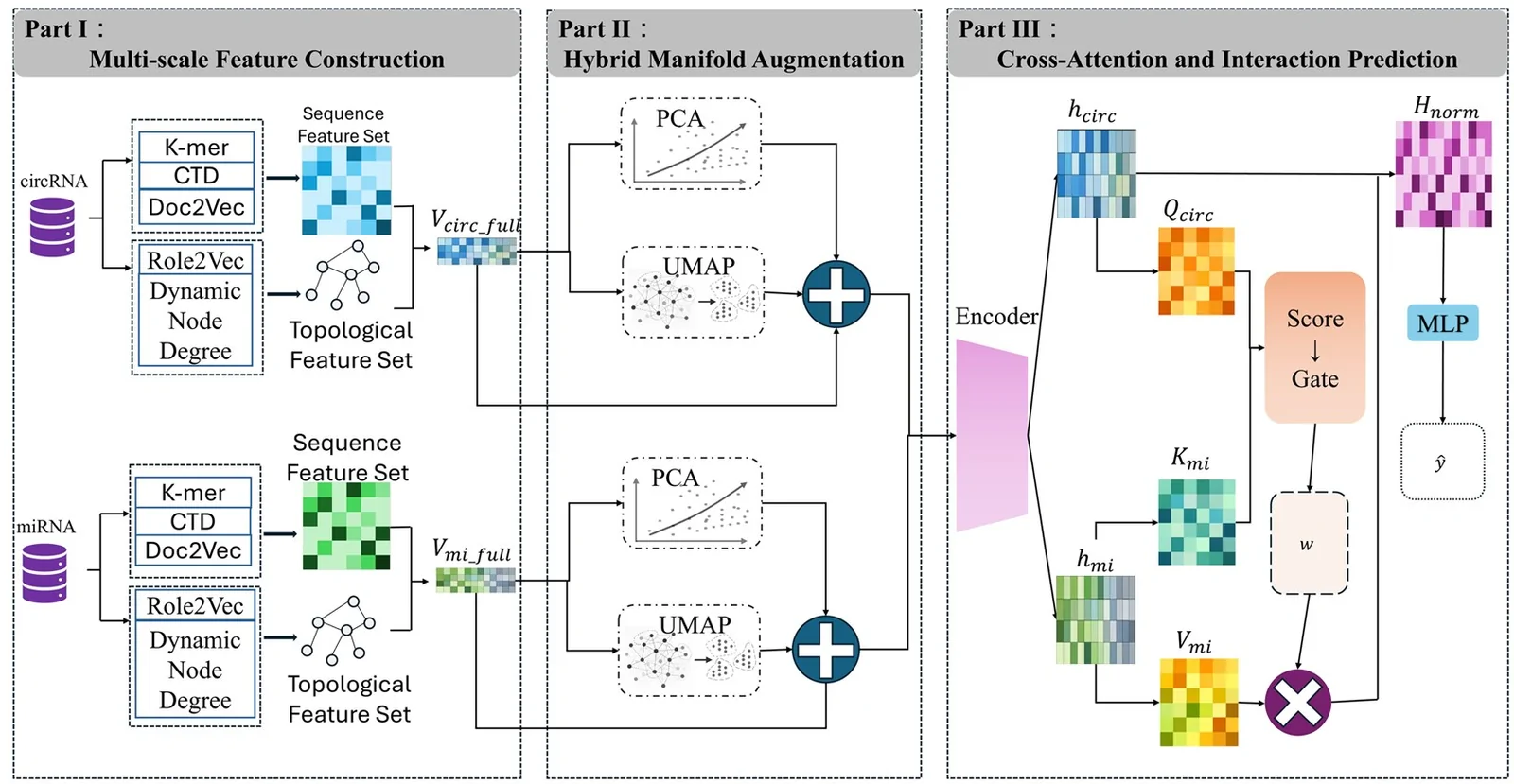
The new framework of the model. Part I: Multi-scale Feature Descriptor Construction, which computes diverse static and dynamic features to provide multi-level initial representations. Part II: Hybrid Manifold Augmentation, where global linear and local nonlinear manifold structures are extracted using PCA and UMAP, and enhanced representations are constructed by combining sequence features and topological features. Finally, they are mapped to high-order implicit embeddings through a deep encoder. Part III: Cross-Attention and Interaction Prediction. This module controls the intensity of miRNA injection into circRNA through a scoring mechanism, constructs the final features, and inputs them into MLP to complete the prediction function.

To overcome these limitations, we propose HMA-GCA which integrates hybrid manifold augmentation with a gated cross-attention mechanism (structure is shown in Fig. 1). HMA-GCA first constructs multi-scale feature descriptors by combining sequence-derived and topological features. It then applies PCA and UMAP to capture both global linear structure and local nonlinear manifold geometry, enriching the feature space while preserving intrinsic data organization. A channel-wise gated cross-attention module computes interaction scores via element-wise product across feature dimensions, enabling fine-grained correlation modeling and dynamically controlling the information flow from miRNA to circRNA. The augmented and cross-attended features are then fused for final prediction. Extensive experiments on three benchmark datasets demonstrate that HMA-GCA achieves competitive performance compared to state-of-the-art methods. Ablation studies validate the essential contribution of each module, while SHAP analysis identifies the most influential feature types-revealing that sequence-derived features and topological similarities play dominant roles in prediction. Furthermore, case studies combined with GO and KEGG enrichment analyses show that top-ranked predictions are supported by literature evidence or exhibit significant functional associations, underscoring the model’s capacity to uncover biologically meaningful and interpretable interactions.

## 2 Methods

### 2.1 Datasets

We selected three datasets from various sources to test the model under the new framework. These datasets were obtained from circBank (Peng *et al*. 2025), BGF-CMA (Guo *et al*. 2024), and the CMI (Wang *et al*. 2023) data extracted by individuals, and were respectively labeled as Dataset 1, Dataset 2, and Dataset 3. The specific indicators of the datasets are shown in Table 1. The number of interaction pairs included were 9589 (2115 ciRNAs and 821 miRNAs), 9905 (2346 ciRNAs and 926 miRNAs), 20208 (3569 ciRNAs and 1152 miRNAs).

**Table 1:** Details of circRNA-miRNA interaction.

Datasets	circRNAs	miRNAs	Interactions
Dataset1	2115	821	9589
Dataset2	2346	926	9905
Dataset3	3569	1152	20208

### 2.2 Multi-scale feature descriptor construction

Since the dual-channel attention mechanism determines correlations based on attention scores, sufficient and informative features are required to support this process. Sequence features, including K-mer (Zhu *et al*. 2023), CTD (Tong and Liu 2019) and Doc2Vec (Lau and Baldwin 2016), are used to capture intrinsic molecular characteristics. In addition, topological features—including similarity aggregation, Role2Vec (Li *et al*. 2023), Dynamic Degree Centrality and Node Degree are employed to describe the relationships of molecules within the global network context.

#### 2.2.1 Sequence features

Sequence features capture the intrinsic biological properties of molecules. So by integrating these sequence features, the properties of RNA can be roughly depicted. In this study, K-mer frequency was used to capture local composition of the sequences, where 1-mer to 4-mer features were extracted to generate a 340-dimensional vector. CTD Descriptors were employed to characterize physicochemical properties, resulting in a 30-dimensional vector. Doc2Vec was utilized to obtain latent representations, and the embedding dimension was set to 256. In addition, similarity features were incorporated to complement the sequence features. All the aforementioned sequence-related features were concatenated to obtain the initial sequence descriptors $v_{\mathrm{seq}\_\mathrm{circ}}$ or $v_{\mathrm{seq}\_mi}$ which corresponding respectively to miRNA and circRNA.

#### 2.2.2 Topological features

After extracting the intrinsic characteristics from RNA sequence features, topological features are introduced to capture global properties. Role2Vec is used to characterize the structural roles of RNAs in the overall network context, resulting in a 256-dimensional embedding. Node degree dynamically calculates node connectivity based on the adjacency matrix of the training set. Suppose the adjacency matrix is defined as $A_{\mathrm{train}}$ and The total number of circRNAs and miRNAs is defined as $N_{\mathrm{circ}}$ and $N_{mi}$ respectively. Then, the degrees of the i-th circRNA(1) and the j-th miRNA(2) can be obtained as follows:


(1) $$v_{\deg}^{\left(i\right)}=\sum_{j=1}^{N_{mi}}A_{\mathrm{train}}(j,i)$$

(2) $$v_{\deg}^{\left(j\right)}=\sum_{j=1}^{N_{\mathrm{circ}}}A_{\mathrm{train}}(j,i)$$

In addition, neighborhood proximity descriptors are employed to capture local clustering information in the feature space. Let the cosine similarity between two points in the feature space be denoted as $\mathrm{Sim}(V_{i},V_{j})$ and assume that each RNA has K most similar neighboring molecules. Then, based on this, the mean similarity (1) and peak similarity (2) can be calculated as follows:


(3) $$v_{\mathrm{mean}}^{\left(i\right)}=\frac{1}{K}\sum_{j=1}^{N_{mi}}\mathrm{Sim}(V_{i},V_{j})$$

(4) $$v_{\mathrm{peak}}^{\left(j\right)}=\underset{j\in \Omega_{i}}{\max}\left\{\mathrm{Sim}(V_{i},V_{j})\right\}$$

To distinguish the parameters corresponding to miRNA and circRNA, subscripts are introduced to the above parameters, resulting in:


$$V_{\deg\_\mathrm{circ}},V_{\deg\_mi},V_{\mathrm{mean}\_\mathrm{circ}},V_{\mathrm{mean}\_mi},V_{\mathrm{peak}\_\mathrm{circ}},V_{\mathrm{peak}\_mi}$$

### 2.3 Hybrid manifold augmentation

In the previous section, we extracted sequence features and topological features, which characterize the individual attributes of RNA and the global status of the network, respectively. We can combine the obtained feature vectors to form a complete representations $V_{\mathrm{full}\_\mathrm{circ}},V_{\mathrm{full}\_mi}$. Next, dimensionality reduction is performed on the high-dimensional features to extract key features and explore the intrinsic manifold structure. Therefore, we introduce PCA and UMAP to achieve this objective.

#### 2.3.1 PCA

Principal Component Analysis (PCA) is a linear dimensionality reduction method. The specific operation is to transform potentially related variables into a set of linearly uncorrelated variables through orthogonal transformation. In this study, a feature vector describing the global linear distribution is defined as Φ. For circRNAs and miRNAs, there are $\Phi_{\mathrm{circ}}$ and $\Phi_{mi}$ respectively. For the previously obtained PCA optimal parameter 32, we thus obtain


(5) $$\Phi_{\mathrm{circ}}=\mathrm{PCA}(V_{\mathrm{full}\_\mathrm{circ}},32\;)$$

(6) $$\Phi_{mi}=\mathrm{PCA}(V_{\mathrm{full}\_mi},32\;)$$

From this, the most representative linear components within the high-dimensional full set of features can be extracted.

#### 2.3.2 UMAP

Uniform Manifold Approximation and Projection (UMAP) is employed to reveal complex non-linear local manifolds. It can capture subtle clustering features while maintaining the domain relationships among data points in the feature vector space. In this study, the characteristics of the local manifold are defined as $\psi_{\mathrm{circ}},\psi_{mi}$. Then based on the function and the parameters 8 we previously obtained we can get:


(7) $$\psi_{\mathrm{circ}}=\mathrm{UMAP}(V_{\mathrm{full}\_\mathrm{circ}},8)$$

(8) $$\psi_{mi}=\mathrm{UMAP}(V_{\mathrm{full}\_mi},8)$$

At this stage, two enhanced feature vectors calculated by PCA and UMAP are available. These vectors are horizontally concatenated (Ramachandram and Taylor 2017, Moon *et al*. 2019) with the previously obtained sequence feature vectors and topological feature vectors to construct the manifold representations $V_{\mathrm{final}\_\mathrm{circ}}$ and $V_{\mathrm{final}\_mi}$, which simultaneously contain the original feature information and the enhanced information. Since the parameters of circRNA and miRNA are always calculated separately, a dual-path encoder is constructed to avoid feature interference (Chen *et al.* 2021, Chopra *et al.* 2005). The two encoders have symmetrical structures but independent parameters. Taking the circRNA encoder $\varepsilon_{\mathrm{circ}}$ as an example. Project $V_{\mathrm{full}\_\mathrm{circ}}$ onto an implicit dimension using the fully connected layer. Introduce the BN layer to accelerate convergence and improve the generalization ability of the model. Construct the ReLU function: σ(x)=max(0,x) and adopt the dropout strategy with p=0.3 to enhance the robustness of the model. Let $W_{\mathrm{circ}}$ represent the weight matrix of the encoder and $b_{\mathrm{circ}}$ represent the bias term of the encoder. Then, we can obtain the final embedding vector $h_{\mathrm{circ}}$:


(9) $$h_{\mathrm{circ}}=\mathrm{Dropout}(\sigma (BN(W_{\mathrm{circ}}V_{\mathrm{final}\_\mathrm{circ}}+b_{\mathrm{circ}})))$$

The same as $\varepsilon_{mi}$.

### 2.4 Cross-attention and interaction prediction

After obtaining the high-order manifold embeddings $h_{\mathrm{circ}}$ and $h_{mi}$, a cross-attention mechanism is introduced to further capture the complex interactions between circRNAs and miRNAs. This mechanism consists of four steps, which can be summarized as Mapping, Scoring, Injection, Fusion-Prediction.

#### 2.4.1 Interaction space mapping

To measure the correlation between circRNA and miRNA on a unified semantic scale, we need to adopt a non-shared weight mechanism to project the previously obtained $h_{\mathrm{circ}}$ or $h_{mi}$ into a dedicated interaction space, thereby obtaining several key vectors for cross-attention computation. Accordingly, the following important vectors can be obtained:


(10) $$Q_{\mathrm{circ}}=h_{\mathrm{circ}}W_{Q}+b_{Q}$$

(11) $$K_{mi}=h_{mi}W_{K}+b_{K}$$

(12) $$V_{mi}=h_{mi}W_{V}+b_{V}$$

Among the above three vectors, $Q_{\mathrm{circ}}$ represents the search tendency of the current circRNA node, $K_{mi}$ is used to construct an index for miRNA, extracting the features in miRNA that can be recognized by external matching, and $V_{mi}$ is used to encapsulate the content semantics of miRNA after determining the association. $W_{Q}\;W_{K}\;W_{V}$ used here represents the weight matrix, and $b_{Q}\;b_{K}\;b_{V}$ represent the bias vector. Based on this, the features of heterogeneous entities are transformed into corresponding queries, keys, and values expressions.

#### 2.4.2 Channel-wise interaction scoring

After completing the mapping section, the model quantifies the matching degree between circRNAs and miRNAs. To this end, the correlation between the two is evaluated from a fine-grained feature perspective. The traditional attention mechanism usually uses the Scaled Dot-product method to measure similarity, which is typically expressed as follows:


(13) $$\mathrm{Attention}(Q,K)=\mathrm{softmax}(\frac{QK^{T}}{\sqrt{d}})$$

This method captures the similarity of vector projections in the global context, but may overlook fine-grained similarities on specific feature channels. Therefore, it is slightly modified to avoid missing similarities on key dimensions.


(14) $$\mathrm{Score}=\frac{\sum_{i=1}^{d}\left(Q_{\mathrm{circ},i}\cdot K_{mi,i}\right)}{\sqrt{d}}$$

The term $Q_{\mathrm{circ},i}\cdot K_{mi,i}$ represents the Hadamard product of the matrix, which yields the values for each channel. These values are then summed up to obtain the similarity measure, which takes into account the similarity relationships across all channels. This similarity relationship essentially represents the correlation. Therefore, the obtained values can be used to quantify the degree of correlation.

#### 2.4.3 Gated information injection

Previously, a quantitative score measuring the correlation between circRNA and miRNA was obtained. This score is then used to guide the incorporation of semantic information from miRNA into the feature representation of circRNA. The obtained Score can be transformed into weight values through the sigmoid activation function. The obtained Score can be transformed into weight value w through the sigmoid activation function. The range of w is (0,1). The higher the score value, the closer it gets to 1, which means the greater the possibility of association between RNA molecules. Then, using the weight w, the previously obtained vector $V_{mi}$ is processed to obtain an enhanced feature term $h_{\mathrm{att}}=w\cdot V_{mi}$. In this way, we can determine whether to incorporate the features based on the previously obtained w to complete the feature completion.

#### 2.4.4 Final feature fusion and association prediction

Now all required features have been obtained. The feature vectors are then fused by concatenating the previously obtained vectors and applying LayerNorm to eliminate magnitude differences. The resulting feature $H_{\mathrm{norm}}$ will use for prediction:


(15) $$H_{\mathrm{norm}}=\mathrm{LayerNorm}(H_{\mathrm{circ}}⊕h_{\mathrm{att}}$$

Finally, $H_{\mathrm{norm}}$ is fed into an MLP to obtain the probability p of the existence of the association.

### 2.5 Experimental setup

We evaluated the proposed HMA-GCA framework on three benchmark datasets. The known CMIs were considered as positive samples and labeled as 1. The unverified CMIs were considered negative samples and labeled as 0. In order to make the number of positive and negative samples match, randomly select the number of negative samples to match the number of positive samples (Zhang and Fan 2017).

For the dataset, we adopt an interaction-pair level split to ensure that identical circRNA–miRNA pairs do not appear in both the training and test sets. This setup guarantees that model evaluation is conducted on previously unseen interactions.

The model was trained with fixed parameter settings to ensure reproducibility. The random seed was set to 42, and the hidden dimension was set to 256. The batch size was set to 256. The learning rate was set to $5\times 10^{-4}$, and the weight decay was set to $5\times 10^{-5}$. The maximum number of training epochs was set to 50, with an early stopping patience of 8 epochs. In addition, gradient clipping was applied with a maximum norm of 5.0 to improve the stability of training.

We evaluated the proposed HMA-GCA framework on three benchmark datasets (detailed in Table 1) using 5‑fold cross‑validation. Model performance was assessed using six metrics: area under the receiver operating characteristic curve (AUC), area under the precision‑recall curve (AUPR), accuracy (ACC), F1‑score, recall, and precision (Dao *et al*. 2025, Riasat *et al*. 2026). All experiments were repeated five times to ensure statistical robustness.

## 3 Results

### 3.1 Cross-validation performance

**Figure 2:**
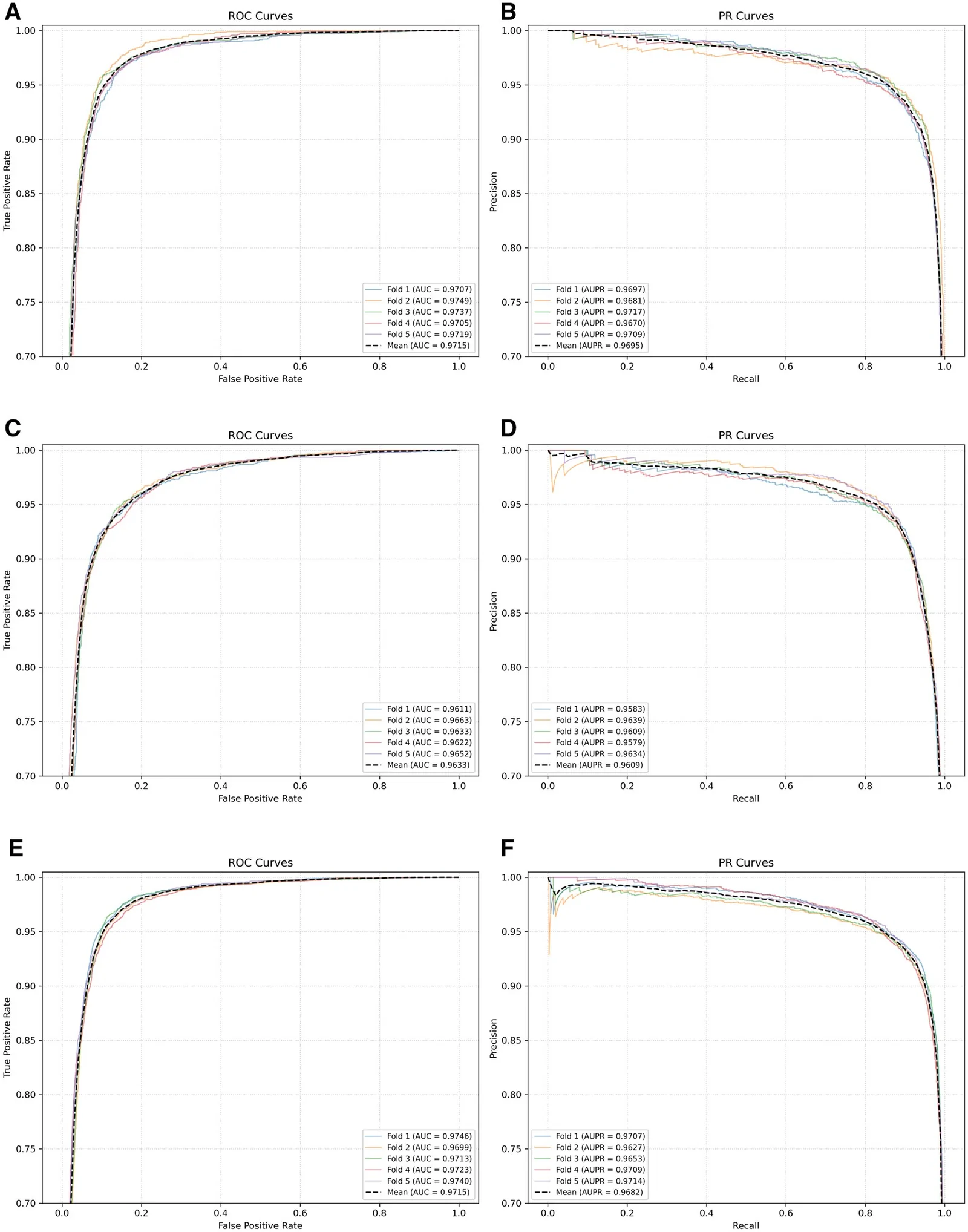
Results of the five-fold cross-validation.

Based on the experimental setup described above, we evaluated the cross-validation performance of HMA-GCA on the three datasets. As shown in Fig. 2, panels A, C, and E present the ROC curves on Datasets 1, 2, and 3, respectively, while panels B, D, and F show the corresponding PR curves. In all three datasets, the ROC curves of different folds are close to the upper-left region, indicating that the model can effectively distinguish positive and negative circRNA–miRNA interaction samples. Meanwhile, the PR curves remain at a relatively high precision level over a wide range of recall values, suggesting that the model maintains stable predictive performance under different fold divisions.

### 3.2 Parameter sensitivity analysis

**Figure 3:**
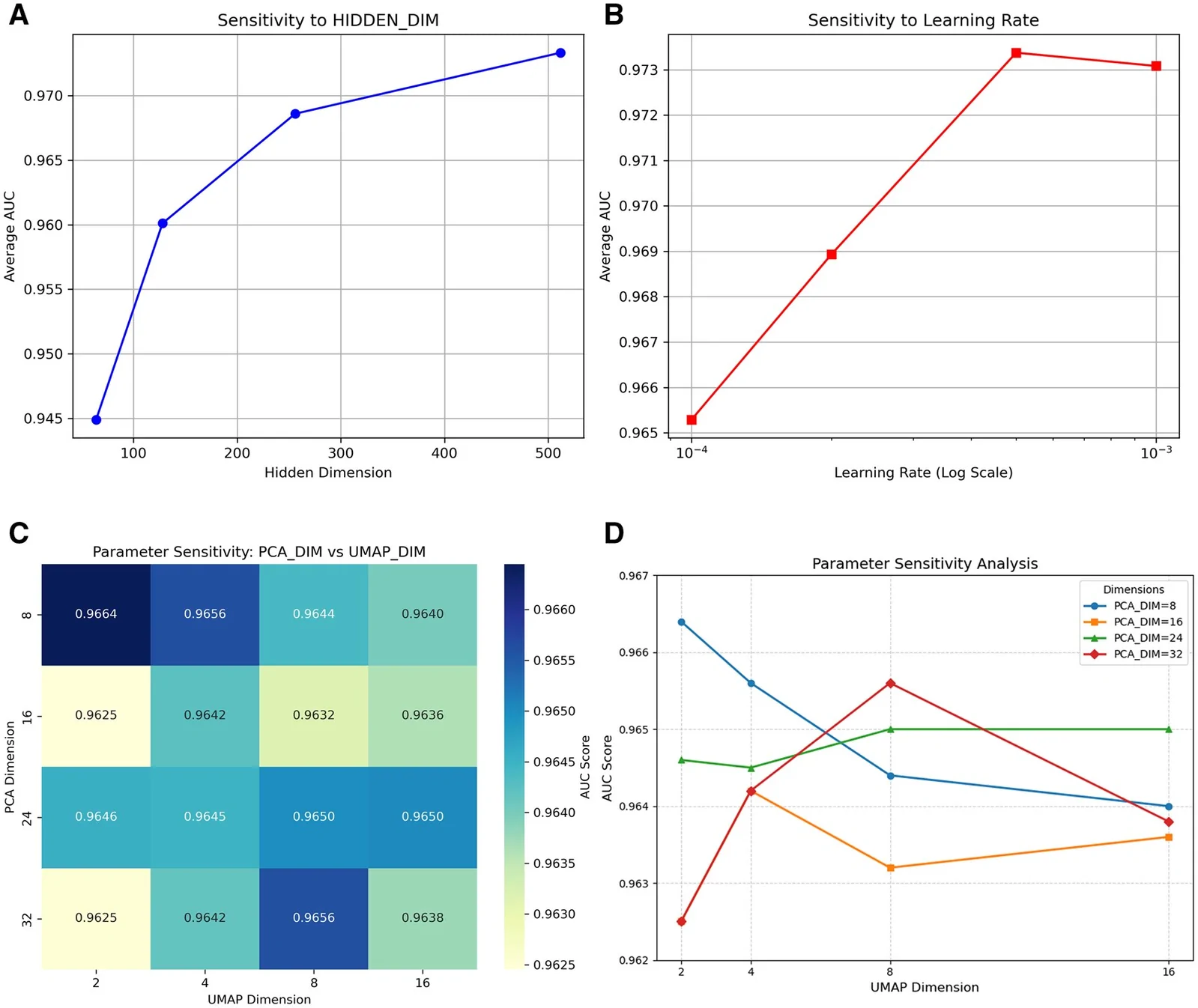
(A) Sensitivity of Hidden Dimension. (B) Sensitivity of Learning Rate. (C) PCA and UMAP Sensitivity Heatmap. (D) PCA and UMAP Sensitivity Line Chart.

Due to the varying sensitivity of the model under different configurations, we adjusted two crucial hyperparameters of the model: the embedding dimension (di) and the learning rate (LR), in order to find the optimal performance situation. Hyperparameter search is conducted using the parallel control variable method. Specifically, the learning rate (LR) is set to $2\times 10^{-4}$ and the optimal di is sought; then, when di is fixed at 256, the optimal LR is determined. After 5-fold cross-validation, we get the specific assessment data and presented it in a visual form as shown in Fig. 3A and B.

### 3.3 Impact of manifold augmentation on feature space

In feature extraction, we have employed PCA and UMAP, and their parameters have a significant impact on the final feature extraction effect. Therefore, we still need to complete the parameter sensitivity experiments of PCA and UMAP. We can see the result in Fig. 3C and D. From the experiments, we find the best combination is PCA_DIM = 8 and UMAP_DIM = 2. However, in this case, the information loss is huge and essentially cannot bring improvement to the model. Therefore, a suboptimal combination is chosen: PCA_DIM = 32 and UMAP_DIM = 8.

**Figure 4:**
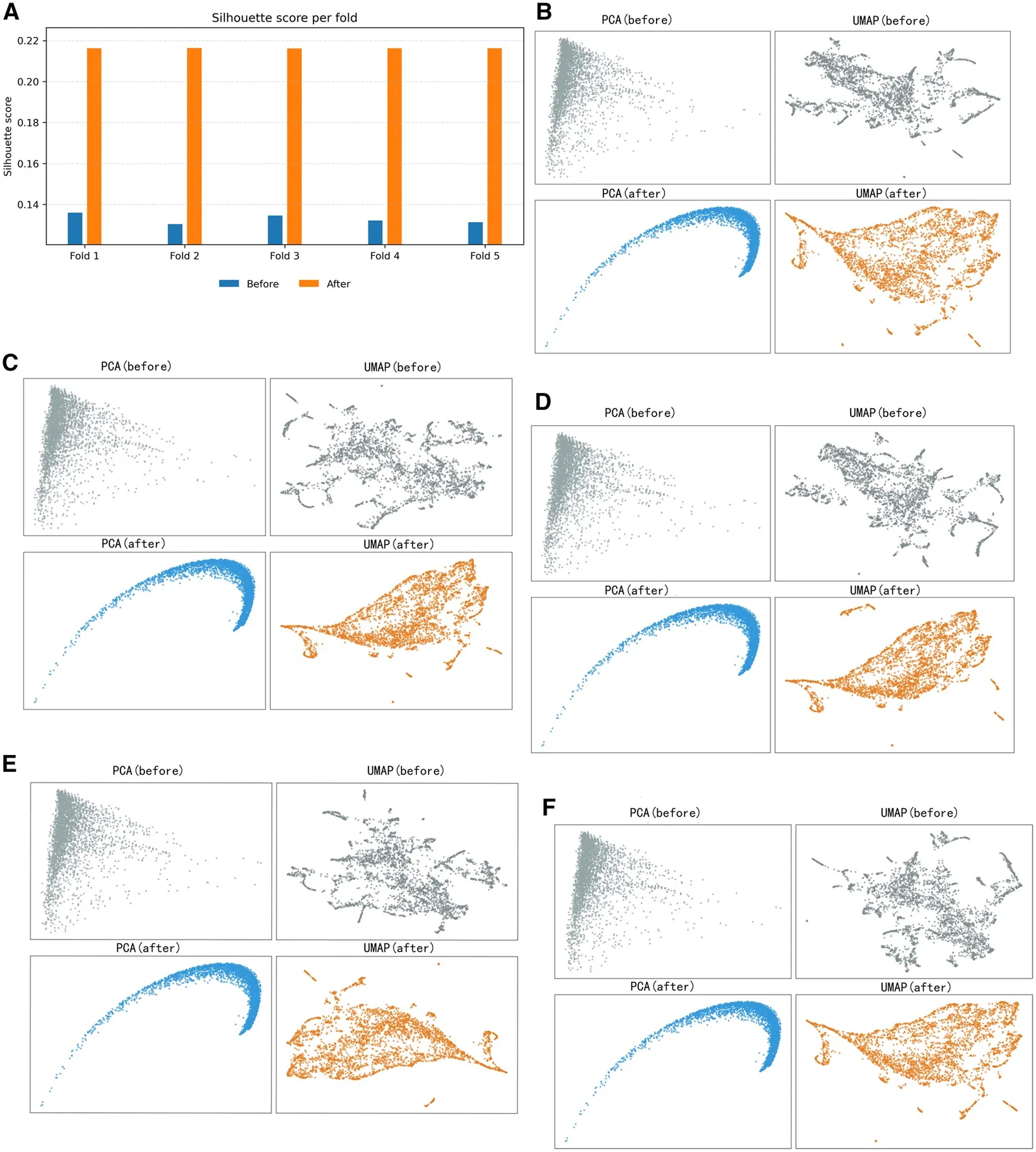
(A) Comparison of silhouette coefficients before and after PCA and UMAP processing on Dataset 3. (B–F) Feature distribution visualizations across the five cross-validation folds.

Take dataset 3, which has the largest number of data samples, as an example, after the processing by PCA and UMAP, the silhouette coefficient has been improved, as shown in Fig. 4A. Figure 4B–F specifically illustrate the changes in feature distribution in the five-fold. PCA integrates the distribution of features into an arc-shaped trajectory, and UMAP further clusters the possible hidden correlations, helping for subsequent predictions.

**Figure 5:**
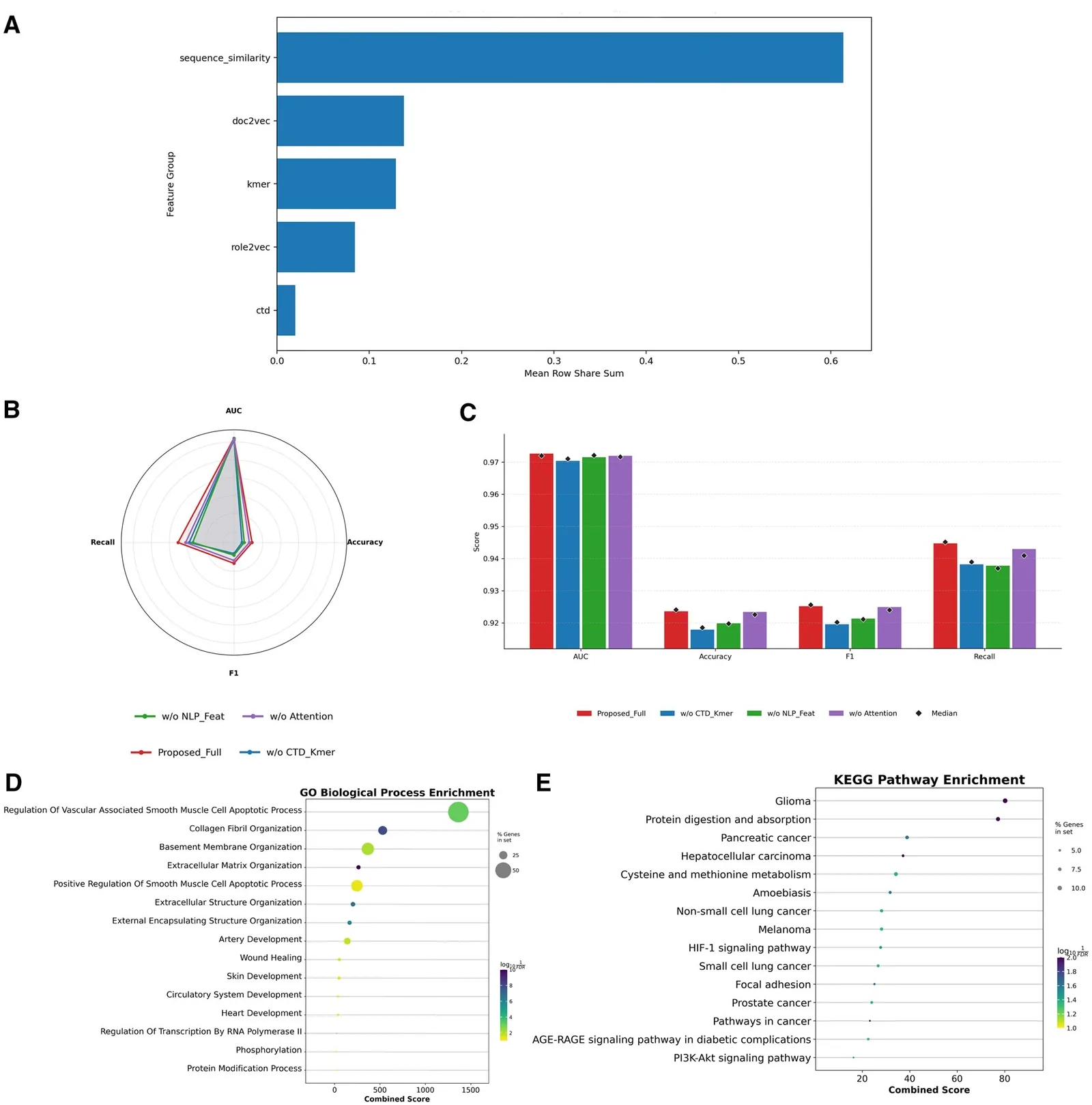
(A) Feature contribution analysis. (B) Radar chart showing Ablation experiments. (C) Line chart showing Ablation experiments. (D) GO biological process enrichment. (E) KEGG pathway enrichment.

Since the processed feature input model cannot specifically distinguish which feature is more effective, we conducted SHAP analysis on the original features that were not processed by PCA and UMAP to identify the input features with high contribution. We summed up the SHAP values obtained from various features and obtained a total table as shown in Fig. 5A. Then, we selected the top 30 features proportionally to specifically examine which features have a high contribution to the prediction. The results are shown in Table 2.

**Table 2:** The top 30 SHAP values.

No	Feature	SHAP	No	Feature	SHAP
1	circ_ctd_3	15.587	16	circ_sequence_similarity_75	6.439
2	circ_doc2vec_29	11.576	17	circ_sequence_similarity_107	6.592
3	circ_doc2vec_23	13.916	18	circ_sequence_similarity_19	3.813
4	circ_doc2vec_5	7.396	19	circ_sequence_similarity_156	5.029
5	circ_doc2vec_30	7.855	20	circ_sequence_similarity_5	4.169
6	circ_kmer_3	8.467	21	circ_sequence_similarity_0	5.442
7	circ_kmer_6	9.758	22	circ_sequence_similarity_84	7.463
8	circ_kmer_1	5.387	23	circ_sequence_similarity_41	6.373
9	circ_kmer_8	6.243	24	circ_sequence_similarity_36	5.165
10	circ_role2vec_18	5.776	25	circ_sequence_similarity_167	5.824
11	circ_role2vec_8	6.630	26	circ_sequence_similarity_303	3.539
12	circ_sequence_similarity_113	5.601	27	circ_sequence_similarity_195	2.586
13	circ_sequence_similarity_6	2.959	28	circ_sequence_similarity_62	4.698
14	circ_sequence_similarity_191	3.203	29	circ_sequence_similarity_86	3.995
15	circ_sequence_similarity_14	4.781	30	circ_sequence_similarity_326	3.360

### 3.4 Comparison with state-of-the-art methods

We compared HMA-GCA with two representative state-of-the-art methods: AMHMDA (Ning *et al*. 2023) and MUSCLE (Ji *et al*. 2024). These two methods were selected because they represent two relevant categories of computational strategies for association prediction. AMHMDA focuses on heterogeneous network-based association modeling, while MUSCLE emphasizes multi-scale feature learning and attention-based feature fusion. Therefore, they provide meaningful references for evaluating the effectiveness of the topological information, multi-scale feature representation, and attention-guided interaction modeling used in HMA-GCA. Table 3 summarizes the comparative results across all three datasets. Our framework consistently outperformed both baselines on all evaluation metrics. Specifically, on Dataset 3, HMA-GCA achieved an AUC of 0.9731 ± 0.0026, F1 of 0.9264 ± 0.0012, ACC of 0.9248 ± 0.0042, and Recall of 0.9473 ± 0.0061, representing improvements of approximately 8.1–12.7% over AMHMDA and 18.0–24.6% over MUSCLE. The relatively lower performance of MUSCLE may be attributed to its multi-scale feature design, which appears less generalizable to datasets with heterogeneous statistical properties (Guo *et al*. 2024, Huang *et al*. 2024, 2024).

**Table 3:** The results of different methods on three datasets.

Datasets	Methods	AUC	F1	ACC	Recall
Dataset1	AMHMDA	0.8869 ± 0.0147	0.7937 ± 0.0349	0.8021 ± 0.0220	0.7723 ± 0.0880
	MUSCLE	0.8755 ± 0.0075	0.8073 ± 0.0052	0.8074 ± 0.0052	0.8071 ± 0.0053
	**OUR**	**0.9713 ± 0.0013**	**0.9256 ± 0.0020**	**0.9237 ± 0.0019**	**0.9499 ± 0.0048**
Dataset2	AMHMDA	0.9030 ± 0.0122	0.8174 ± 0.0230	0.8262 ± 0.0230	0.7863 ± 0.0747
	MUSCLE	0.8319 ± 0.0055	0.7672 ± 0.0044	0.7173 ± 0.0057	0.7517 ± 0.0084
	**OUR**	**0.9630 ± 0.0021**	**0.9098 ± 0.0050**	**0.9088 ± 0.0054**	**0.9198 ± 0.0067**
Dataset3	AMHMDA	0.9003 ± 0.0041	0.8262 ± 0.0024	0.8208 ± 0.0017	0.8521 ± 0.0053
	MUSCLE	0.8244 ± 0.0070	0.7600 ± 0.0059	0.7595 ± 0.0059	0.7600 ± 0.0072
	**OUR**	**0.9731 ± 0.0026**	**0.9264 ± 0.0012**	**0.9248 ± 0.0042**	**0.9473 ± 0.0061**

Bold values indicate the best performance for each evaluation metric.

### 3.5 Ablation study of the feature learning

To verify that the new framework is indeed superior, this section compares the original model with the three variant models on dataset 3. The three variant models are introduced as follows:

- *“w/o CTD_Kmer”* remove the basic biological characteristic information and rely solely on the high-level semantics.
- *“w/o NLP_Feat”* remove the high-level semantics extracted by NLP and rely solely on the basic biological feature information.
- *“w/o Attention”* remove the dual-channel attention mechanism and instead use the direct concatenation of feature vectors

The points in the Fig. 5B and C represent the multiple results of the five-fold cross-validation. To reduce the influence of extreme cases on the overall results, the median values are plotted. In Fig. 5C, the bars represent the mean values, while the black dots indicate the median values, providing a more comprehensive view of the performance distribution. The charts show that when basic biological information or high-level semantic features are absent, the overall performance of the model declines significantly. Meanwhile, after incorporating the multi-channel attention mechanism, the performance of the model is further improved. These results indicate that introducing a dynamic interaction mechanism is superior to the traditional static fusion strategy.

### 3.6 Case study

In order to verify the predictive effect of the new model on the interaction between circRNAs and miRNAs, we used dataset 3 as the training object, obtaining the best model for each fold under the 5-fold scenario. Subsequently, we used these models to score the unknown relationships, selecting the top ten with the highest average scores and listing them. Then, we verified the correctness of the predicted correlations by consulting the literature. The results are shown in Table 4. As can be seen from the results, a portion of the high-score correlations predicted by the model have indeed been experimentally verified.

**Table 4:** The top 10 interaction identified by HMA-GCA.

circRNAs	miRNAs	Evidence
hsa_circ_0001116	hsa-miR-1184	PMID:35284630
hsa_circ_0041116	hsa-miR-6751-5p	unconfirmed
hsa_circ_0041089	hsa-miR-1229-5p	unconfirmed
hsa_circ_0041116	hsa-miR-1229-5p	unconfirmed
hsa_circ_0068783	hsa-miR-93-5p	PMID:33585208
hsa_circ_0019689	hsa-miR-212-5p	PMID:37870214
hsa_circ_0041891	hsa-miR-378a-3p	PMID:35093879
hsa_circ_0020490	hsa-miR-3182	unconfirmed
hsa_circ_0013876	hsa-miR-331-3p	unconfirmed
hsa_circ_0044520	hsa-miR-29c-3p	unconfirmed

The remaining unverified relationships do not necessarily indicate that the model made a wrong prediction. We will verify this through enrichment analysis. First, we obtain the host genes of the unverified relationship pairs from circBase. The host genes of the several groups of circRNAs in the unverified relationship pairs are TCF25, MKI67, NBPF10, and COL1A1. Then, we search for the high-scoring target genes corresponding to the miRNAs in the relationship pairs on miRDB. We select the target genes with a score higher than 80 as the input and conduct GO and KEGG analyses respectively. The final results are shown in Fig. 5D and E. The analysis of GO indicates that there is indeed a certain enrichment in the predicted relationships, which implies that the model’s predictions do not merely capture noise but rather identify potentially valid associations. The analysis of KEGG further supports this conclusion.

## 4 Discussion and conclusion

In this study, we proposed HMA-GCA, a framework that integrates hybrid manifold augmentation with a channel-wise gated cross-attention mechanism for CMI prediction. By enriching multi-scale sequence and topological features with global linear and local nonlinear manifold representations, the model captures both geometric structure and fine-grained cross-modal interactions, leading to more accurate and biologically meaningful predictions. Experiments on three benchmark datasets show that HMA-GCA consistently outperforms state-of-the-art methods across all metrics. Ablation studies confirm the necessity of each component, while SHAP analysis identifies key contributing features. Case studies reveal that top-ranked predictions are supported by literature or exhibit significant functional enrichment.

Despite these advances, several limitations remain. First, incorporating PCA and UMAP reduces model interpretability; post-hoc tracing to original features is lost, restricting SHAP analysis to pre-manifold features. Second, random negative sampling may include undiscovered positives, potentially biasing training-evidenced by score variability across cross-validation folds. To mitigate the potential impact of mislabeled negative samples, we adopt random negative sampling and five-fold cross-validation to reduce the variability introduced by a single sampling and to enhance the stability of training and evaluation. Third, the model exhibits popularity bias, favoring well-studied genes (e.g. TCF25) while under-scoring some verified interactions, suggesting over-learning on high‑degree nodes.

Future work should address these issues through: (i) developing post-hoc interpretability methods for manifold-transformed features; (ii) adopting robust negative sampling strategies (e.g., PU learning); (iii) incorporating bias-aware regularization to mitigate over-fitting on frequent nodes; and (iv) integrating tissue-specific or temporal expression data to capture context-dependent interactions.

## Data Availability

The datasets underlying this article are publicly available in the GitHub repository at https://github.com/Lixunwind/Prediction-circ-mi-by-Gate/tree/main/datasets.
